# Heterologous expression of *mtf* and *mtc* genes of *Pseudanabaena foetida* var. *intermedia* is sufficient to produce 2-methylisoborneol in *Escherichia coli*

**DOI:** 10.1128/spectrum.02561-23

**Published:** 2023-09-21

**Authors:** Kaushalya Dayarathne, Toshiki Ishikawa, Satoru Watanabe, Yuuma Ishikawa, Kadeer Aikeranmu, Hina Kitagawaa, Natsumi Komatsubara, Masatoshi Yamaguchi, Maki Kawai-Yamada

**Affiliations:** 1 Graduate School of Science and Engineering, Saitama University, Shimo-Okubo, Sakura-ku, Saitama-city, Saitama, Japan; 2 Department of Bioscience, Tokyo University of Agriculture, 1-chōme-1-1 Sakuragaoka, Setagaya-city, Tokyo, Japan; 3 Institute for Molecular Physiology, Heinrich-Heine-Universität, Cluster of Excellence on Plant Sciences (CEPLAS), Düsseldorf, Germany; University of Minnesota Twin Cities, St. Paul, Minnesota, USA

**Keywords:** 2-MIB, *Pseudanabaena foetida *var. *intermedia*, *mtf*, *mtc*

## Abstract

Microbial volatile metabolite 2-methylisoborneol (2-MIB) causes odor and taste issues in drinking water, making it unappealing for human consumption. It has been suggested that 2-MIB biosynthesis consists of two main steps, namely, methylation of geranyl diphosphate into 2-methyl geranyl diphosphate by geranyl diphosphate methyl transferase (GPPMT) and subsequent cyclization into 2-MIB by 2-MIB synthase (MIBS). *Pseudanabaena foetida* var. *intermedia* is a 2-MIB-producing cyanobacterium whose GPPMT and MIBS enzymes are encoded by adjacent *mtf* and *mtc* genes*.* The present study identified a 2-MIB-related gene cluster composed of *cnb*A, *mtf*, *mtc*, and *cnb*B genes in *P*. *foetida* var. *intermedia*. The two homologous cyclic nucleotide-binding protein genes, *cnb*A and *cnb*B, were detected adjacent to the *mtf* and *mtc* genes, respectively. The nucleotide sequence of the *cnb*A-*mtf-mtc-cnb*B gene cluster showed 99.55% identity with 2-MIB synthesis-associated gene cluster of *Pseudanabaena* sp. dqh15. RT-PCR results revealed that *mtf* and *mtc* genes are co-expressed, while *cnb*A and *cnb*B genes are expressed independently in *P*. *foetida* var. *intermedia*. To investigate whether only *mtf* and *mtc* genes are sufficient for 2-MIB synthesis, the two-gene unit (*mtf-mtc*) was introduced into *Escherichia coli* strain JM109 via overexpression vector pYS1C. Gas chromatograph-mass spectrometry results showed that the *E. coli* strain transformed with *mtf-mtc* was able to produce 2-MIB. The intracellular 2-MIB level in *P*. *foetida* var. *intermedia* was higher than the extracellular 2-MIB level, while the transformed *E. coli* strain showed an opposite trend. Growth inhibition was observed in the 2-MIB-producing transformed *E. coli* strain.

**IMPORTANCE:**

Contamination of drinking water with odiferous microbial metabolite 2-MIB is a worldwide concern. Removal of 2-MIB from drinking water burdens the water purification process. Therefore, it is important to search for alternative methods, such as suppressing the production of 2-MIB by aquatic microorganisms. For that, it is necessary to expand the current knowledge about the mechanism of 2-MIB synthesis at the genetic level. This study revealed that *mtf* and *mtc* genes of the 2-MIB-related gene cluster are transcribed as a single unit in *P*. *foetida* var. *intermedia*, and the expression of both *mtf* and *mtc* genes is essential and sufficient for 2-MIB synthesis in *E. coli* heterologous gene expression system.

## INTRODUCTION

The production of odiferous microbial metabolites in freshwater reservoirs has become a major environmental concern worldwide. It dramatically reduces the quality of drinking water. 2-Methylisoborneol (2-MIB) and geosmin are the primary microbial metabolites responsible for the unpleasant musty odor and taste in drinking water and freshwater fish (1, 2). Cyanobacteria*,* actinomycetes, myxobacteria, and fungi are the dominant producers of 2-MIB and geosmin-like odiferous metabolites in water bodies (3 – 5). Any toxic effects of geosmin or 2-MIB on humans have not yet been reported. However, since these compounds are detectable even at concentrations as low as 10 ng/L, consumers are reluctant to drink the contaminated water (6). Powdered activated carbon treatment and ozonation are currently used to remove these taste and odor compounds from drinking water (7, 8). Given the high cost and unexpected safety risks associated with these methods, there is a significant demand for alternative methods, such as suppressing the production of odiferous metabolites by aquatic microorganisms.

2-MIB is a volatile methylated monoterpene alcohol (9). A previous feeding study in myxobacteria elucidated that 2-MIB biosynthesis involves two steps (10). In the first step, geranyl diphosphate (GPP), the universal precursor of monoterpenes, is methylated to 2-methyl GPP by geranyl diphosphate methyl transferase (GPPMT), where S-adenosyl-L-methionine (SAM) serves as the donor of the methyl group. In the second step, 2-methyl GPP is cyclized to 2-MIB by 2-MIB synthase (MIBS) in the presence of Mg^2+^. Wang et al. (11) identified GPPMT and MIBS encoding genes (*mtf* and *mic*, respectively) from cyanobacteria strains *Pseudanabaena* sp. dqh15 and *Planktothricoides raciborskii* CHAB 333. They have described that those two genes are arranged in the order of *mtf* followed by the *mic* and located between two homologous cyclic nucleotide-binding protein genes (*cnb*). Furthermore, Komatsu et al. (12), according to their bioinformatics analysis, assumed that the gene encoding MIBS (*mtc*) involved in 2-MIB biosynthesis forms an operon with GPPMT (*mtf*) gene in bacteria. However, no experimental evidence is available to elucidate whether these genes are co-expressed as operon genes during 2-MIB production.

*Pseudanabaena foetida* var. *intermedia* (formerly identified as *Pseudanabaena galeata*) is a 2-MIB producing filamentous, non-heterocystous freshwater cyanobacteria first isolated from Nagoya castle, Nagoya, Aichi Prefecture, Japan. It is commonly found in freshwater bodies such as ponds and lakes (13). NIES-512 is a strain of *P*. *foetida* var. *intermedia*, which serves as a model strain in 2-MIB-related studies. In our previous study, we detected the GPPMT and MIBS encoding genes (*pgmtf* and *pgmtc*, respectively) in NIES-512 (in that study, NIES-512 was mentioned as *Pseudanabaena galeata*). We found that the temperature conditions of the culture affect the expression level of those two genes (14).

Although previous studies have described the biochemical reaction of 2-MIB synthesis and related gene sequences, the complete genetic mechanism of 2-MIB synthesis in cyanobacteria has yet to be clearly understood. The main objectives of this study were to investigate how 2-MIB-related genes are transcribed in NIES-512 and to determine which genes are essential for 2-MIB synthesis.

## MATERIALS AND METHODS

### Cyanobacteria strain and growth conditions

Freshwater 2-MIB-producing cyanobacteria *Pseudanabaena foetida* var. *intermedia* strain NIES-512 was obtained from the microbial culture collection at National Institute for Environmental Studies (NIES), Tsukuba, Japan. NIES-512 was cultured in liquid BG-11 medium (pH 8.0) (15), and static cultures were maintained at 20°C under a 12-h photoperiod with 20-µmol photons/m^2^/s of light intensity.

### DNA extraction

NIES-512 cells were collected from 1 mL of liquid culture (OD_730_ = 0.5) by centrifugation at 8,000 *g* at 4°C for 5 min. The cell pellet was washed with distilled water and resuspended in 200 µL of 10-mM EDTA (pH 8.0). Then, the cell suspension was subjected to three times repeated freeze-thawing cycles (−80°C for 3 min and 60°C for 2 min). After centrifugation at 8,000 *g* at 4°C for 2 min, the supernatant was collected for genomic PCR analysis.

### Identification of 2-MIB-related gene cluster and bioinformatic analysis

2MIB-related gene cluster from NIES-512 genomic DNA was amplified by primers (cnbA-F: 5′-ATGACAGAAAATCTAGATTCTAATCAGC-3′ and cnbB-R: 5′-CTACCGCCCGATCTCG ACATCCTCGAGA-3′) designed based on the nucleotide sequence information of *Pseudanabaena* sp. dqh15 reported by Wang et al. (11) (GenBank accession number: HQ830028.1). The amplified gene fragment was sequenced using CEQ8000 (Beckman Coulter, USA), and the sequence data was submitted to the DNA Data Bank of Japan (DDBJ) (accession number: LC765370). Open reading frames (ORFs) of the nucleotide sequence were identified by NCBI ORF finder (http://www.ncbi.nlm.nih.gov/gorf/gorf.html). The nucleotide sequence of the 2-MIB-related gene cluster in NIES-512 was analyzed and aligned using Molecular Evolutionary Genetics Analysis software version 11 and nucleotide-nucleotide Basic Local Alignment Search Tool nucleotide program (https://blast.ncbi.nlm.nih.gov/Blast.cgi?PROGRAM=blastn).

### RNA extraction and cDNA synthesis

Cells were collected from 20 mL of NIES-512 liquid culture (OD_730_ = 0.5). The cell pellet was ground to a powder under freezing conditions using liquid nitrogen, and RNA was extracted by guanidinium thiocyanate-phenol-chloroform RNA extraction method (16). The extracted total RNA was purified using RNeasy Plant Mini Kit and RNase-Free DNase set (QIAGEN, Germany) according to the instructions given by the manufacturer. The purity of the purified total RNA was tested by obtaining the A260:A280 absorption ratio (1.8–2.0). Then, cDNA was synthesized using a high-capacity cDNA reverse transcription Super-Script III Kit (Invitrogen, USA) according to the instructions given by the manufacturer.

### PCR analysis

Primers were designed based on the nucleotide sequence information of the *cnb*A-*mtf-mtc-cnb*B gene fragment of the NIES-512 strain obtained from this study. Primers used for the PCR analysis in this study are listed in Table 1. First, the individual *cnb*A, *mtf*, *mtc*, and *cnb*B genes in NIES-512 were tested by RT-PCR to detect the respective gene transcripts. The amplification of each gene transcript was performed using a thermal cycler (Applied Biosystems 2720) in 10 µL of total reaction volume containing 1 µL of cDNA, 5 µL of Quick taq HS dye mix (Takara, Japan), and 0.5 µL of each 20-µM forward and reverse primers. The thermocycler program included initial denaturation at 94°C for 2 min, followed by 30 cycles of denaturation at 94°C for 30 s, annealing at 58°C for 30 s, and extension at 68°C for 1 min. To examine the association of *cnb*A, *mtf*, *mtc*, and *cnb*B genes with each other during the expression, RT-PCR was conducted to amplify six possible transcripts (*mtf-mtc*, *cnb*A-*mtf-mtc-cnb*B, *cnb*A-*mtf*, *cnb*A-*mtf-mtc*, *mtc-cnb*B, and *mtf-mtc-cnb*B). PCR amplification of the *cnb*A-*mtf-mtc-cnb*B fragment was performed in 10 µL of total reaction volume containing 1 µL of cDNA, 5 µL of KOD One dye mix (Takara), and 0.2 µL of each 20-µM forward and reverse primers. The thermocycler program consisted of 30 cycles of denaturation at 98°C for 10 s, annealing at 60°C for 5 s, and extension at 68°C for 30 s. PCR amplification of the other five fragments was done using 5 µL of Quick taq HS dye mix in 10 µL of total reaction volume containing 1 µL of cDNA and 0.2 µL of each 20-µM forward and reverse primers (thermal cycler program: initial denaturation at 94°C for 2 min, followed by 35 cycles of denaturation at 94°C for 30 s, annealing at 58°C for 30 s, and extension at 68°C for 5 min). Genome PCR was also conducted with the same conditions replacing cDNA with genomic DNA to amplify the above six gene fragments. Amplified RT-PCR and genome PCR products were subjected to agarose gel electrophoresis and detected after ethidium bromide staining.

**TABLE 1:** Primers for PCR

PCR target	Forward primer	Reverse primer
*cnb*A	5′-ATGACAGAAAATCTAGATTCTAATCAGC-3′	5′-CTAGCGTCCGAGTTCGACATCTTCGAGT-3′
*cnb*B	5′-ATGACCCAAGACTTTAACTCCCATGGGC-3′	5′-CTACCGCCCGATCTCGACATCCTCGAGA-3′
*mtf*	5′-ATGTCAACGCCCCAAACTATCACTGCCG-3′	5′-TTACCGAATGATGCGGTCAGCAACG AT-3′
*mtc*	5′-ATGAAAGATACCAACTTGGATAATACG-3′	5′-TTAGGCTAGTGATTGTGAATCTGGCTG-3′
*cnb*A-*mtf-mtc-cnb*B	5′-ATGACAGAAAATCTAGATTCTAATCAGC-3′	5′-CTACCGCCCGATCTCGACATCCTCGAGA-3′
*cnb*A-*mtf-mtc*	5′-ATGACAGAAAATCTAGATTCTAATCAGC-3′	5′-TTAGGCTAGTGATTGTGAATCTGGCTG-3′
*mtf-mtc-cnb*B	5′-ATGTCAACGCCCCAAACTATCACTGCCG-3′	5′-CTACCGCCCGATCTCGACATCCTCGAGA-3′
*cnb*A-*mtf*	5′-ATGACAGAAAATCTAGATTCTAATCAGC-3′	5′-TTACCGAATGATGCGGTCAGCAACG AT-3′
*mtf-mtc*	5′-ATGTCAACGCCCCAAACTATCACTGCCG-3′	5′-TTAGGCTAGTGATTGTGAATCTGGCTG-3′
*mtc-cnb*B	5′-ATGAAAGATACCAACTTGGATAATACG-3′	5′-CTACCGCCCGATCTCGACATCCTCGAGA-3′

### Construction of plasmids and *Escherichia coli* strains

NIES-512 *mtf* and *mtc* genes were introduced to expression vector pYS1C designed by Sakamaki et al. (17) using In-Fusion HD Cloning Kit (Takara Bio, Japan) to construct pYS1C-mtf, pYS1C-mtc, and pYS1C-mtf-mtc plasmid vectors. Plasmid vector pYS1C-green fluorescent protein (GFP) harboring the gene encoding GFP was used as the negative control. All the plasmid constructs were verified by DNA sequencing. Plasmids were transformed into *E. coli* JM109 by heat shock method, and *E. coli*-pYS1C-GFP, *E. coli*-pYS1C-mtf, *E. coli*-pYS1C-mtc, and *E. coli*-pYS1C-mtf-mtc strains were obtained. Solid LB medium (18) containing 35 µg/L chloramphenicol was used to select the transformed strains.

### 2-MIB analysis by GC-MS

For the analysis of 2-MIB production in transformed *E. coli* strains*,* 50 µL of overnight grown liquid cultures was inoculated to 5 mL of fresh LB medium (containing 35-µg/mL chloramphenicol) and cultured at 37°C under constant shaking at 140 rpm. For the cultures tested under isopropyl β-D-1-thiogalactopyranoside (IPTG) induction, 1 mM IPTG was added to the medium after 2 h of the inoculation. Cells were harvested for the gas chromatography-mass spectrometry (GC-MS) analysis after 5 h of the inoculation. For the analysis of 2-MIB production in NIES-512, cells were collected from 12-day-old cultures. For measuring total 2-MIB levels, OD measured (OD_600_ for *E. coli* and OD_730_ for NIES-512) 1 mL of cell culture was used, while for analyzing intracellular and extracellular 2-MIB levels, cell pellet (resuspended in 1 mL of distilled water) and the supernatant separated from 1 mL of cell culture (by centrifugation at 8,000 *g* at room temperature for 5 min) were used, respectively. Collected 1 mL of the sample was added to a glass tube containing 1 mL of methanol and 1 mL of hexane. Then, the samples were vortexed and centrifuged at 120 *g* for 10 min at room temperature. The hexane extract was used to analyze 2-MIB by GC-MS (Shimadzu GCMS-QP2010) equipped with a TC-70 column (0.25 µm × 0.25 mm × 30 m, GL Science, Japan) using He as the carrier gas at 37.7 cm/s of linear velocity and the temperature program: held at 60°C for 3 min, 20°C/min to 200°C, held for 5 min. 2-MIB was detected by total ion scanning or selected ion monitoring (SIM) mode using *m*/*z* 95 (predominantly used for quantification), 108, and 69 (used for qualification) by EI (70 eV). See Fig. S1 for the MS spectrum obtained by the 2-MIB standard (FUJIFILM Wako Chemicals, USA). SIM was used due to the higher sensitivity necessary for detecting low levels of 2-MIB in biological samples. 2-MIB content was calculated using the standard curve, and the obtained value was normalized with the OD value of the respective culture sample used for the GC-MS analysis to determine the 2-MIB contents in 1 mL of culture per unit OD value.

### Growth analysis

Overnight grown 100-µL cultures of *E. coli*-pYS1C-GFP and *E. coli*-pYS1C-mtf-mtc were separately inoculated to 10 mL of fresh LB medium (containing 35-µg/mL chloramphenicol) with a starting OD of 0.03 at 600 nm and cultured at 37°C under constant shaking at 140 rpm. The growth of the cell cultures was studied by measuring the OD value at 600 nm hourly for 10 h. For the cultures tested under IPTG induction, 1-mM IPTG was added to the medium after 2 h of the inoculation.

### Statistical analysis

Statistical analysis was conducted using the Statistical Package for the Social Sciences (SPSS version 20.0).

## RESULTS

### Gene arrangement and expression of 2-MIB-related genes in NIES-512

**FIG 1:**
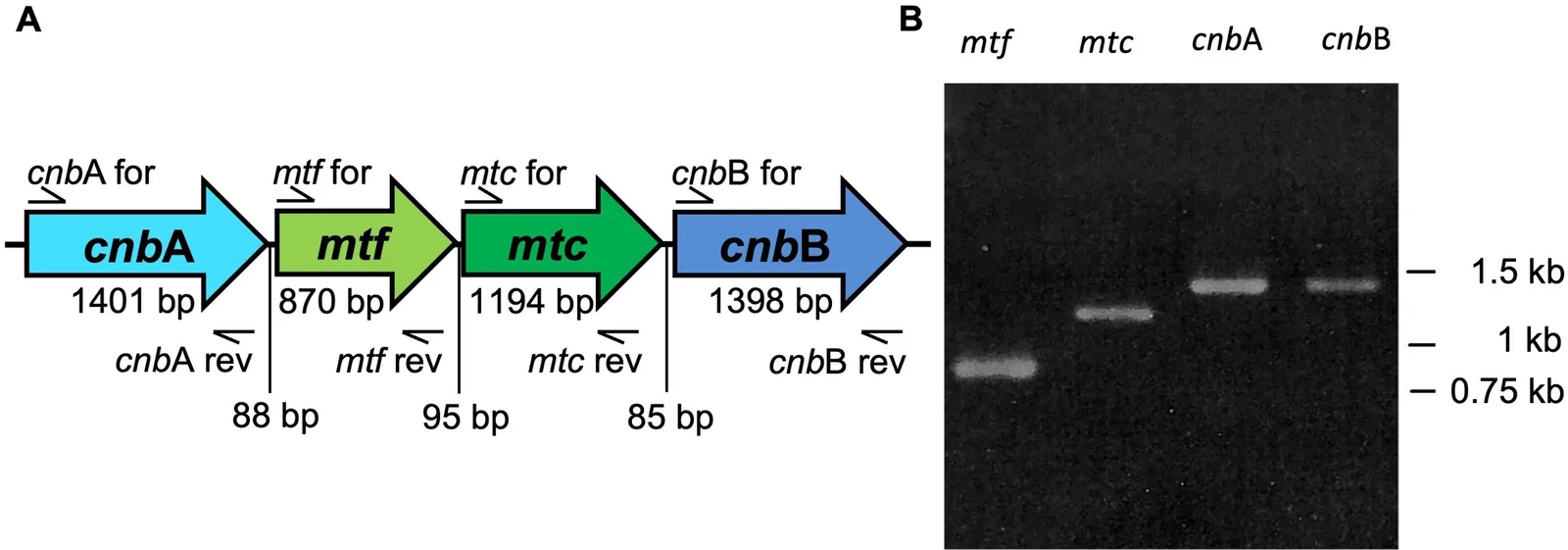
The gene arrangement and RT-PCR amplification of 2-MIB-related genes in NIES-512. (**A**) Arrangement of 2-MIB synthesis-related genes in the NIES-512 genome. The numbers indicate the estimated sizes of each gene and gaps between the genes. Primer pairs shown with each gene (Table 1) were used for RT-PCR amplification. (**B**) Agarose gel electrophoresis results of RT-PCR amplified *mtf*, *mtc*, *cnb*A, and *cnb*B genes from NIES-512. Numbers indicate the band positions of the DNA size marker. The experiment was repeated two times independently with similar results.

Complete nucleotide sequence of the 2-MIB-related gene cluster of NIES-512 (DDBJ accession number: LC765370) showed high homology to 2-MIB-related genes of *Pseudanabaena* sp. dqh15 (NCBI accession number: HQ830028.1), *Microcoleus pseudautumnalis* (NCBI accession number: LC486303.1), and *P. raciborskii* (NCBI accession number: HQ830029.1) with 99.55%, 88.99%, and 87.16% of percentage identity values, respectively. Four ORFs with nucleotide lengths of 1,401 base pair (bp), 870 bp, 1,194 bp, and 1,398 bp were detected in the nucleotide sequence of the 2-MIB-related gene cluster of NIES-512. By comparing the gene arrangement and nucleotide sequence of the 2-MIB-related genes of the filamentous cyanobacterial species *Pseudanabaena* sp. dqh15, *P. raciborskii*, and *M. pseudautumnalis* presented by previous studies (11, 19), we revealed that these four open reading frames are, respectively, representing *cnb*A, *mtf*, *mtc*, and *cnb*B genes, and the four genes are arranged in the order of *cnb*A, *mtf*, *mtc*, and *cnb*B in the NIES-512 genome. The size of the gap between *cnb*A and *mtf* genes was 88 bp, whereas the gaps between *mtf* and *mtc* and *mtc* and *cnb*B were 95 bp and 85 bp, respectively (Fig. 1A).

**FIG 2:**
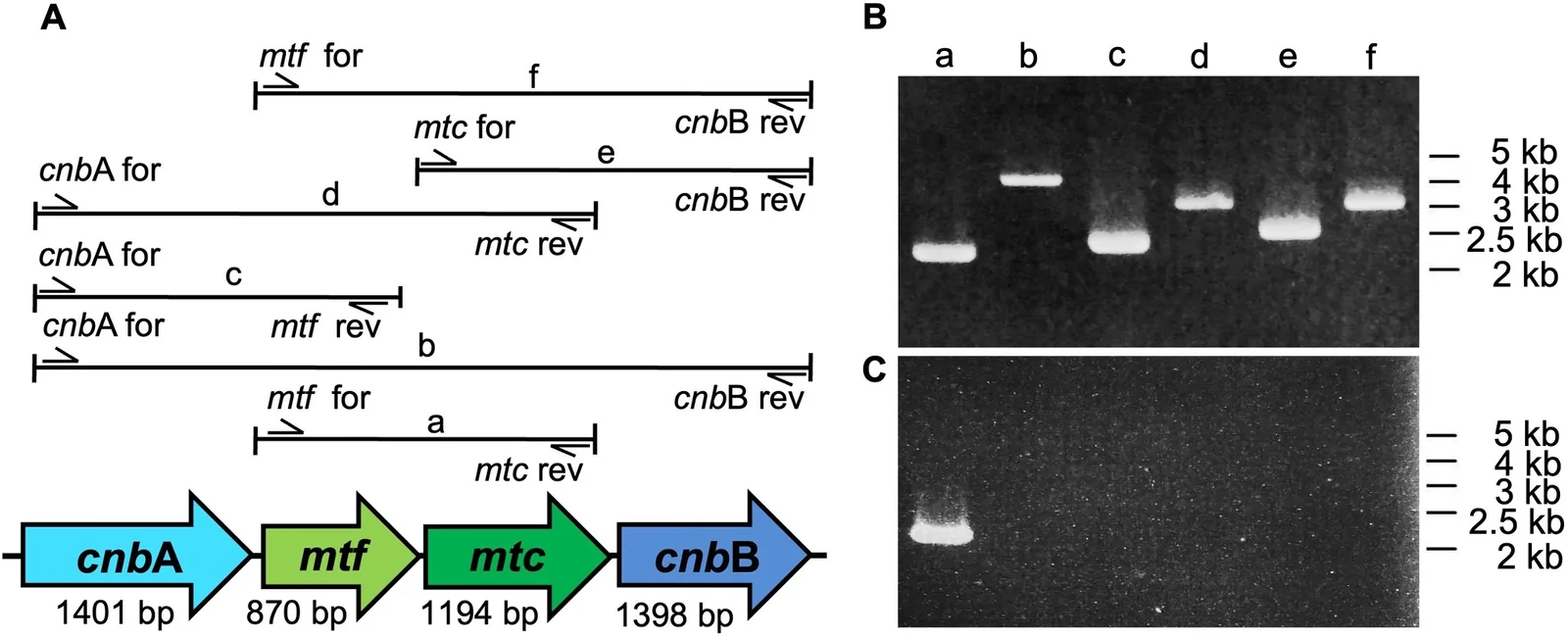
Association between 2-MIB-related genes during the gene expression in NIES-512. (**A**) Possible transcripts from 2-MIB synthesis-related gene cluster in the NIES-512 genome (a, *mtf-mtc*; b, *cnb*A-*mtf-mtc-cnb*B; c, *cnb*A-*mtf*; d, *cnb*A-*mtf-mtc*; e, *mtc-cnb*B; f, *mtf-mtc-cnb*B). Primer pairs indicated with each gene fragment (Table 1) were used for genomic and RT-PCR amplification. (**B**) Agarose gel electrophoresis results of PCR amplified gene fragments (a–f) from NIES-512 genomic DNA. (**C**) Agarose gel electrophoresis results of PCR amplified gene fragments (a–f) from NIES-512 cDNA. Numbers indicate the band positions of the DNA size marker. The experiment was repeated two times independently with similar results.

Given the close arrangement of these *mtf*, *mtc*, and *cnb* genes on the genome of several 2-MIB-producing microbial species, previous studies have assumed that all the above four genes are involved in 2-MIB biosynthesis (11, 19). However, currently, there is a lack of experimental evidence to substantiate this idea. Therefore, to test if all *cnb*A, *mtf*, *mtc*, and *cnb*B genes are expressed in NIES-512, RT-PCR was conducted to amplify four genes independently. The results indicated that all four genes are expressed in NIES-512 (Fig. 1B). To test whether these four genes are expressed as operons, an RT-PCR experiment targeting six possible gene transcripts (a–f) was performed (Fig. 2A). Genome PCR was done as the control experiment to ensure that corresponding primer pairs could amplify each gene fragment (Fig. 2B). However, out of six suspected gene transcripts, only the *mtf-mtc* transcript was detected by RT-PCR results (Fig. 2C). The results indicated that *cnb*A and *cnb*B genes are transcribed independently, and *mtf* and *mtc* genes are transcribed together as a single unit in NIES-512.

### 2-MIB production in *E. coli* strains harboring *mtf* and *mtc* genes

**FIG 3:**
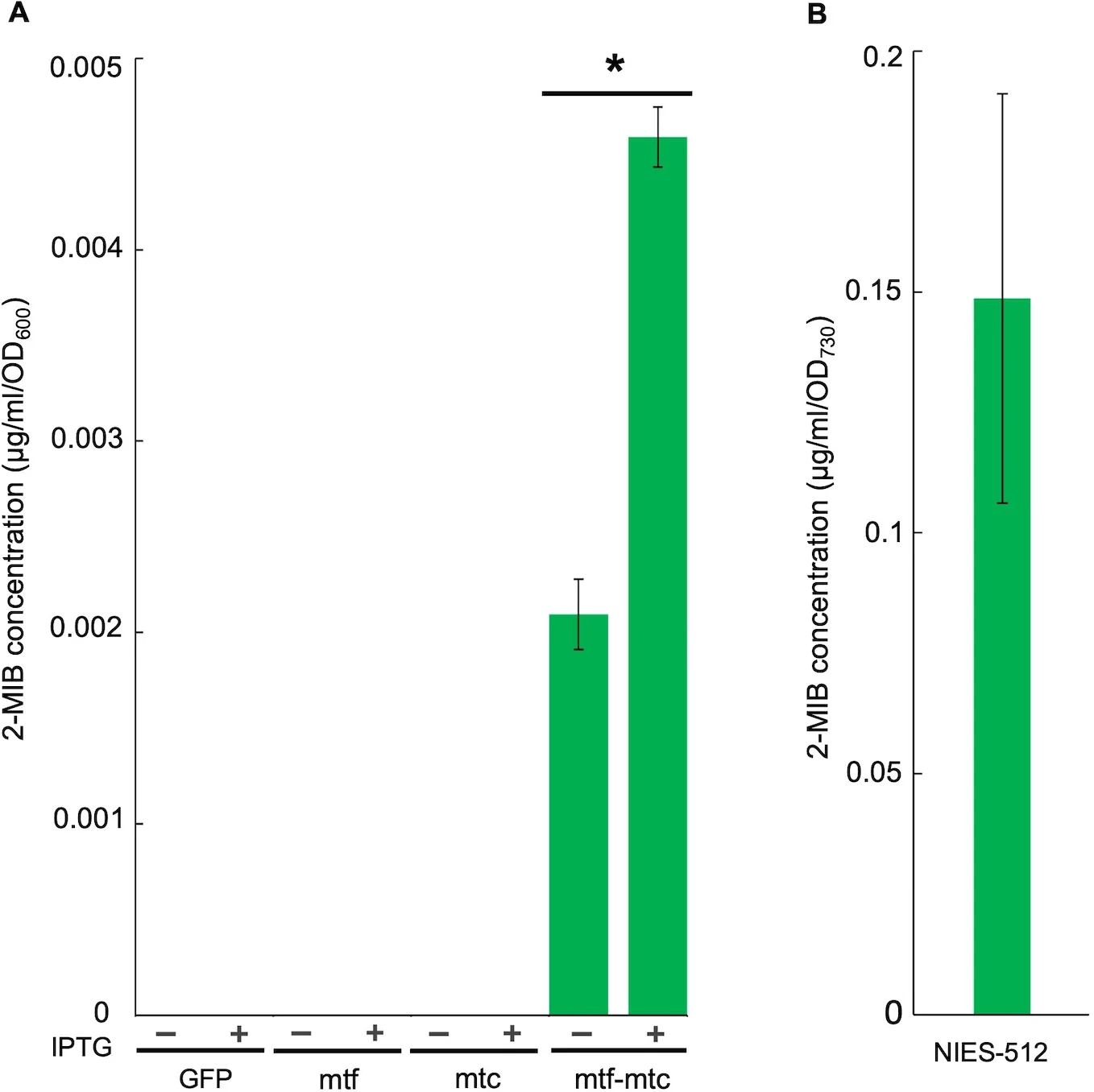
The total 2-MIB production by transgenic *E. coli* strains and NIES-512. (**A**) The total 2-MIB production by *E. coli*-pYS1C-mtf, mtc, and mtf-mtc strains with IPTG induction (+) and without IPTG induction (−). *E. coli*-pYS1C-GFP served as the negative control. (**B**) The total 2-MIB production by NIES-512. 2-MIB levels denoted by * significantly differ at *P* < 0.01 (*t*-test). *n* = 3 (mean ± standard deviation).

To investigate whether the *mtf* and *mtc* genes alone could synthesize 2-MIB, the *mtf* and *mtc* genes individually and as a two-gene unit were introduced into *E. coli* cells. The production of 2-MIB in the presence of *mtf* gene only, *mtc* gene only, and with the *mtf-mtc* unit was studied using *E. coli*-pYS1C-mtf, *E. coli*-pYS1C-mtc, and *E. coli*-pYS1C-mtf-mtc strains, respectively. *E. coli*-pYS1C-GFP strain, which harbors the GFP encoding gene, served as the control strain. Total 2-MIB production (intracellular and extracellular 2-MIB contents) in those *E. coli* strains was studied separately under IPTG-induced and non-induced conditions. IPTG served as the inducer for the *trc* (trp-lac) promotor (a leaky promotor), the promotor used to express gene inserts in the pYS1C vector system. In GC-MS analysis, 2-MIB was detected only from the *E. coli* strain carrying the *mtf-mtc* gene unit (*E. coli*-pYS1C-mtf-mtc). The peak detected in the transformant was validated by comparison with the chemical standard, showing the same retention time and MS fragment pattern (quantitative and qualitative ions in SIM mode), which were completely overlapped as a single peak after spiking the *E. coli* sample with the 2-MIB standard (Fig. S2). In contrast, the *E. coli* strains carrying a single *mtf* or *mtc* gene were not able to produce 2-MIB (Fig. 3A), indicating that both *mtf* and *mtc* genes are involved in the 2-MIB synthesis in *E coli* heterologous gene expression system. The total 2-MIB production by the *E. coli*-pYS1C-mtf-mtc strain under IPTG induction was 0.0045µg/mL/OD_600_, which was approximately twofold higher than the total 2-MIB production by the same strain under zero IPTG induction. The total 2-MIB production in NIES-512 was 0.148 µg/mL/OD_730_ (Fig. 3B).

### Intracellular and extracellular 2-MIB levels in *E. coli* strain harboring *mtf-mtc*

**FIG 4:**
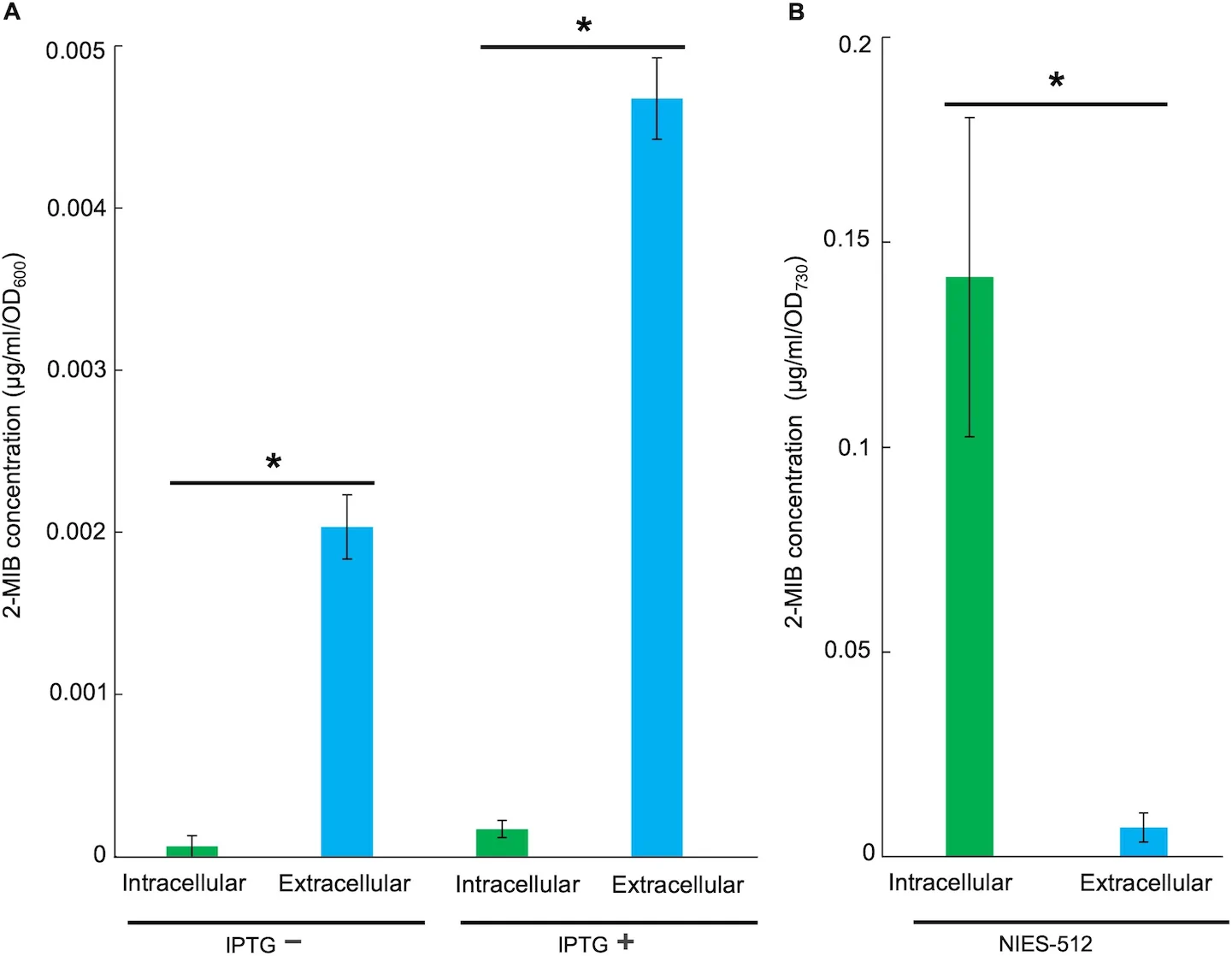
The difference between 2-MIB release and retention by *E. coli* and NIES-512. (**A**) Intracellular and extracellular 2-MIB levels in *E. coli*-pYS1C-mtf-mtc strains cultured without and with IPTG induction. (**B**) Intracellular and extracellular 2-MIB levels in NIES-512. 2-MIB levels denoted by * significantly differ at *P* < 0.01 (*t*-test). *n* = 3 (mean ± standard deviation).

To investigate the difference in cyanobacteria and *E. coli* regarding the release of 2-MIB into the external environment, intracellular and extracellular 2-MIB levels in both the NIES-512 strain and *E. coli*-pYS1C-mtf-mtc strain were examined by GC-MS analysis. In *E. coli*-pYS1C-mtf-mtc, the extracellular 2-MIB level was significantly higher than the intracellular 2-MIB level under IPTG-induced and non-induced conditions (Fig. 4A). Under IPTG-induced conditions, extracellular 2-MIB content exhibited by *E. coli*-pYS1C-mtf-mtc was 0.0046 µg/mL/OD_600_, whereas the intracellular 2-MIB content was only 0.0001 µg/mL/OD_600_. Interestingly, NIES-512 cells showed an opposite trend where the intracellular 2-MIB level (0.14 µg/mL/OD_730_) was higher than the extracellular 2-MIB level (0.007 µg/mL/OD_730_) (Fig. 4B).

### Growth analysis of transformed *E. coli* strains

**FIG 5:**
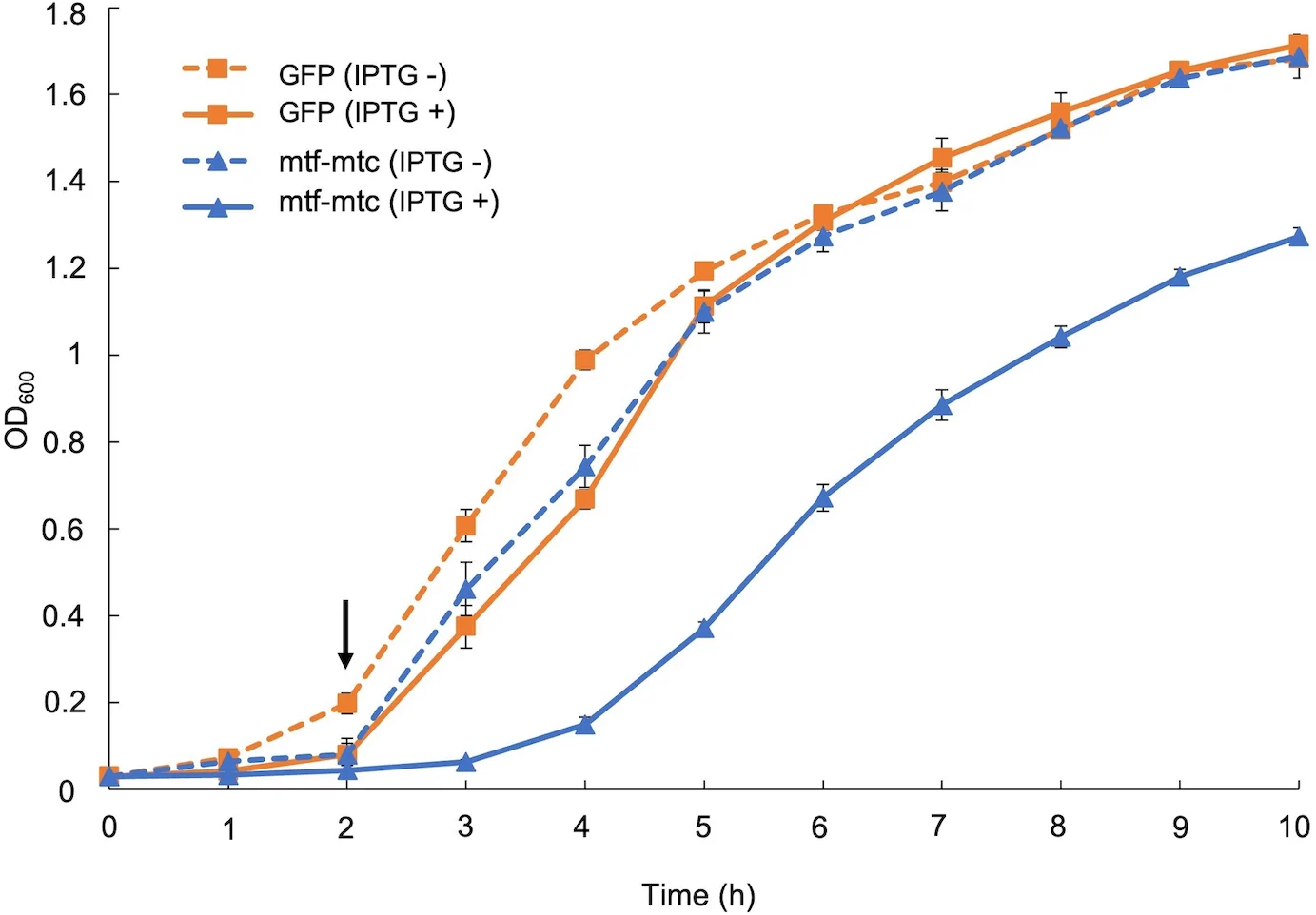
The growth of *E. coli*-pYS1C-mtf-mtc and *E. coli*-pYS1C-GFP strains. The arrow indicates the time point when IPTG was added to the cultures tested under IPTG induction. *n* = 3 (mean ± standard deviation).

The growth of the *E. coli*-pYS1C-mtf-mtc strain was studied under both IPTG-induced and non-induced conditions compared to the growth of the control strain (*E. coli*-pYS1C-GFP) (Fig. 5). The growth of the *E. coli*-pYS1C-mtf-mtc strain was markedly reduced compared to the growth of *E. coli*-pYS1C-GFP under IPTG-induced conditions. In the absence of IPTG induction, the growth of the *E. coli*-pYS1C-mtf-mtc strain was slightly decreased only during the exponential growth phase but then gradually reached the same growth pattern as the control strain.

## DISCUSSION

Although 2-MIB has been identified as an odiferous cyanobacterial metabolite, there is considerable scope for further exploration and investigation of its biosynthesis mechanism in cyanobacteria. The main goal of this study was to investigate how 2-MIB-related genes are expressed in the *P. foetida* var. *intermedia* NIES-512 strain and how the expression of *mtf* and *mtc* genes affects the 2-MIB production.

This study revealed that the gene arrangement of 2-MIB-related genes in the NIES-512 genome is *cnb*A*-mtf-mtc-cnb*B. The high homology and similar gene arrangement of these four genes with other 2-MIB-producing filamentous cyanobacteria (such as *Pseudanabaena* sp. dqh15, *P. raciborskii*, and *M. pseudautumnalis*) speculate the particular importance of all four genes in 2-MIB production. Confirming this idea, our gene expression data demonstrated that all four *mtf*, *mtc, cnb*A, and *cnb*B genes are expressed in NIES-512, whereas *mtf* and *mtc* genes are expressed together as a single unit. Considering the co-expression of *mtf* and *mtc* genes, we suspect that the promotor for expression of the *mtf-mtc* unit could be located at the C-terminal end of the *cnb*A gene in NIES-512. Moreover, the independent expression of *cnb* genes raises the possibility that other regulatory factors might be involved in the expression and coordination of *cnb* genes in cyanobacteria.

In a previous study, genes of 2-MIB-producing soil bacteria *Streptomyces lasaliensi* were used to synthesize 2-MIB *in vitro* by recombinant GPP methyl transferase and 2-MIB synthase in the presence of GPP, SAM, and MgCl_2_ (12). In the present study, we reported *in vivo* synthesis of 2-MIB by introducing the *mtf* and *mtc* genes of the NIES-512 strain into *E. coli* using pYS1C overexpression vector and confirmed that the expression of both *mtf* and *mtc* genes is essential for 2-MIB synthesis in *E. coli*. Interestingly, this result also indicates that the expression of only the *mtf-mtc* gene unit is sufficient to produce 2-MIB in the absence of both *cnb*A and *cnb*B genes under the *trc* overexpression promoter *in E. coli* heterologous gene expression system.

A previous study by Kschowak et al. (20) demonstrated the production of 2-MIB in *E. coli* by the heterologous expression of GPPMT and MIBS genes from streptomyces. In their study, the *E. coli* strain has been modified to produce high GPP (precursor for the 2-MIB) levels by introducing mevalonate pathway genes and GPP synthase genes. *E. coli* natively employs the non-mevalonate pathway for the biosynthesis of isoprenoids, including GPP, which is inherently associated with lower efficiency compared to the mevalonate pathway (21, 22). However, in the present study, GPP produced by the native non-mevalonate pathway in the *E. coli* JM109 strain was used as the precursor for 2-MIB synthesis. Even though the native GPP production in *E. coli* is typically limited (23), the *E. coli*-pYS1C-mtf-mtc strain produced a detectable amount of 2-MIB in the present study.

In Conserved Domain Database (CDD) analysis, two conserved domains, namely, the effector domain of the catabolic gene activator protein (CAP) family of transcription factors and a phage capsid-like protein domain, were identified within protein sequences of both *cnb*A and *cnb*B genes. The CAP family of transcription factors plays a crucial role in regulating gene expression in response to specific environmental signals (24). CAP proteins typically consist of two functional domains: DNA-binding and effector domains. The binding of the effector molecules (cAMP) makes conformational changes in CAP proteins, enhancing their affinity for DNA and promoting gene expression (25). However, as the protein sequences of both *cnb*A and *cnb*B genes showed only the effector domain of CAP proteins, it can be assumed that *cnb* proteins of NIES-512 could not directly bind to DNA and may require the interaction of other cellular factors for the regulation of gene expression. Moreover, the presence of the phage capsid-like protein domain in protein sequences of both *cnb*A and *cnb*B genes of NIES-512 suggests potential roles of cnb proteins similar to viral capsid proteins, such as protein-protein interactions and self-assembly. Based on the proximity of *cnb*A and *cnb*B genes to *mtf* and *mtc* genes, their expression in NIES-512, and the results of CDD analysis, there is a suggestive indication of a potential association between *cnb* genes and 2-MIB synthesis, such as determination and regulation of 2-MIB level produced by the cyanobacteria or controlling the cellular localization of 2-MIB. A previous study also proposed that *cnb* genes may serve as regulators of 2-MIB biosynthesis or physiological responses associated with 2-MIB in cells (11).

In this study, we revealed that intracellular 2-MIB content is higher in NIES-512, while almost all the 2-MIBs produced by *E. coli*-pYS1C-mtf-mtc are released into the extracellular medium. Based on this observation, it can be assumed that NIES-512 cells produce 2-MIB for a particular purpose and store it inside the cells until used. For example, it can be hypothesized that 2-MIB produced by NIES-512 is retained in cells and released upon cell death, where 2-MIB may act as an allelopathic compound to maintain population survival under stressed conditions or may serve as a stress signaling compound within the population to adapt the living receiver cells against the stress condition. A study by Zhang et al. (26) has described the allelopathic effect of 2-MIB produced by *Pseudanabaena* sp. on co-existing cyanobacteria *Microcystis aeruginosa*. In *E. coli*-pYS1C-mtf-mtc strain, almost all the produced 2-MIB has been removed from the cells due to the lack of a mechanism to store the produced 2-MIB inside cells or 2-MIB toxicity.

In this study, significant growth inhibition was observed in 2-MIB-producing *E. coli*-pYS1C-mtf-mtc strain under IPTG-induced conditions, which may be possibly caused due to 2-MIB toxicity. However, even without IPTG induction, the *E. coli*-pYS1C-mtf-mtc strain produced a considerable amount of 2-MIB but showed a relatively slight growth inhibition limited to the exponential growth phase. A possible reason for this observation could be the ability of *E. coli* cells to tolerate 2-MIB toxicity to a certain threshold level by actively excreting the produced 2-MIB without affecting cellular functions. Considering the growth inhibition in the *E. coli*-pYS1C-mtf-mtc strain due to 2-MIB production, we suggest possible toxic effects of 2-MIB against other organisms. Supporting this idea, a recent study also showed that exposure to 2-MIB at a concentration of 42.8 µg/L induced oxidative stress and cell apoptosis in zebrafish larvae (27). Therefore, we emphasize the need for further studies to investigate the toxic or adverse effects of 2-MIB on other aquatic organisms and humans.

In conclusion, the results of this study confirm that *mtf* and *mtc* genes are co-expressed, while *cnb*A and *cnb*B genes are expressed independently in cyanobacteria *P. foetida* var. *intermedia* NIES-512 strain. The expression of both *mtf* and *mtc* genes is essential for 2-MIB synthesis, and these two genes are sufficient to produce 2-MIB in the absence of both *cnb*A and *cnb*B genes under the *trc* overexpression promoter in *E. coli* heterologous gene expression system. Furthermore, this study revealed the specific ability of *P. foetida* var. *intermedia* NIES-512 strain to retain 2-MIB within the cells and the growth inhibitory effect of 2-MIB on the heterologous 2-MIB expressing *E. coli* cells. We suspect cyanobacteria produce 2-MIB as a defense or signaling substance to ensure the survival of the population. Further research is required to elucidate the precise role of *cnb*A and *cnb*B genes in the biosynthesis of 2-MIB, the mechanisms employed by cyanobacteria for intracellular retention of 2-MIB, and the biochemical and physiological implications of suppressing 2-MIB production in cyanobacteria.

## Data Availability

The complete nucleotide sequence of the 2-MIB-related gene cluster of *P*. *foetida* var. *intermedia* identified in this study is available in NCBI with the accession number LC765370.

## SUPPLEMENTAL MATERIAL

Supplemental material spectrum.02561-23-s0001.pdf Fig. S1 and S2.

ASM does not own the copyrights to Supplemental Material that may be linked to, or accessed through, an article. The authors have granted ASM a non-exclusive, world-wide license to publish the Supplemental Material files. Please contact the corresponding author directly for reuse.
