# Supplementary material for: Heterologous expression of mtf and mtc genes of Pseudanabaena foetida var. intermedia is sufficient to produce 2-methylisoborneol in Escherichia coli
Source: Microbiol Spectr. 2023 Sep 21;11(5):e02561-23. doi: 10.1128/spectrum.02561-23 (PMC10580876; doi:10.1128/spectrum.02561-23)
Supplement: Supplemental material — Fig. S1 and S2. [file spectrum.02561-23-s0001.pdf]

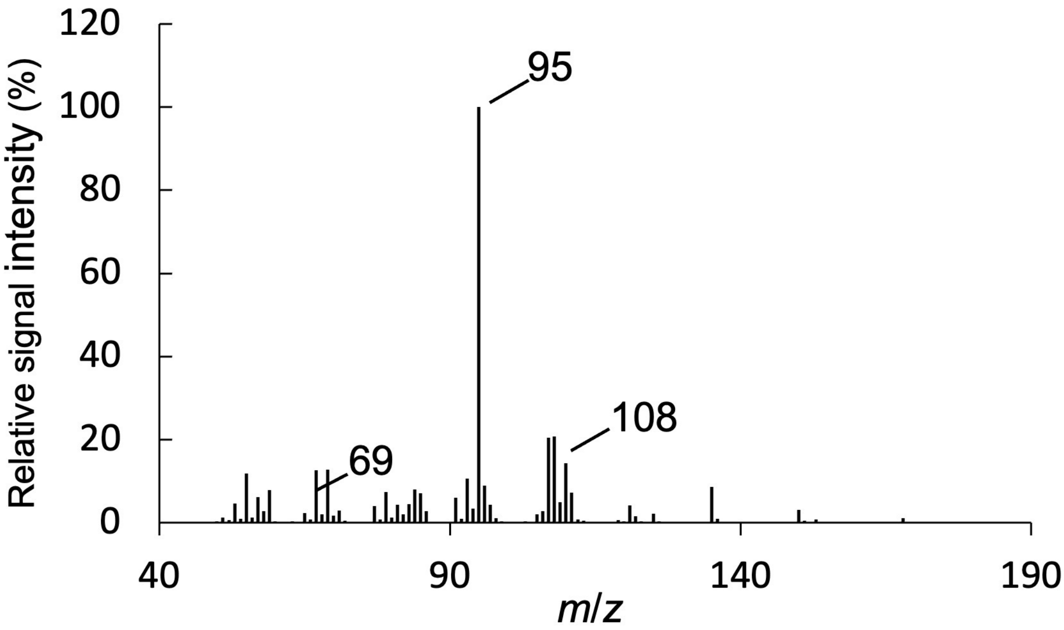

SUPPLEMENTARY FIGURE 1: The MS spectrum of 2-MIB obtained by GC-MS analysis of 2-MIB standard. The major fragments at *m*/*z* 95, 108 and 69 were used for quantitative and qualitative ions in the selected ion monitoring of 2-MIB extracted from biological samples.

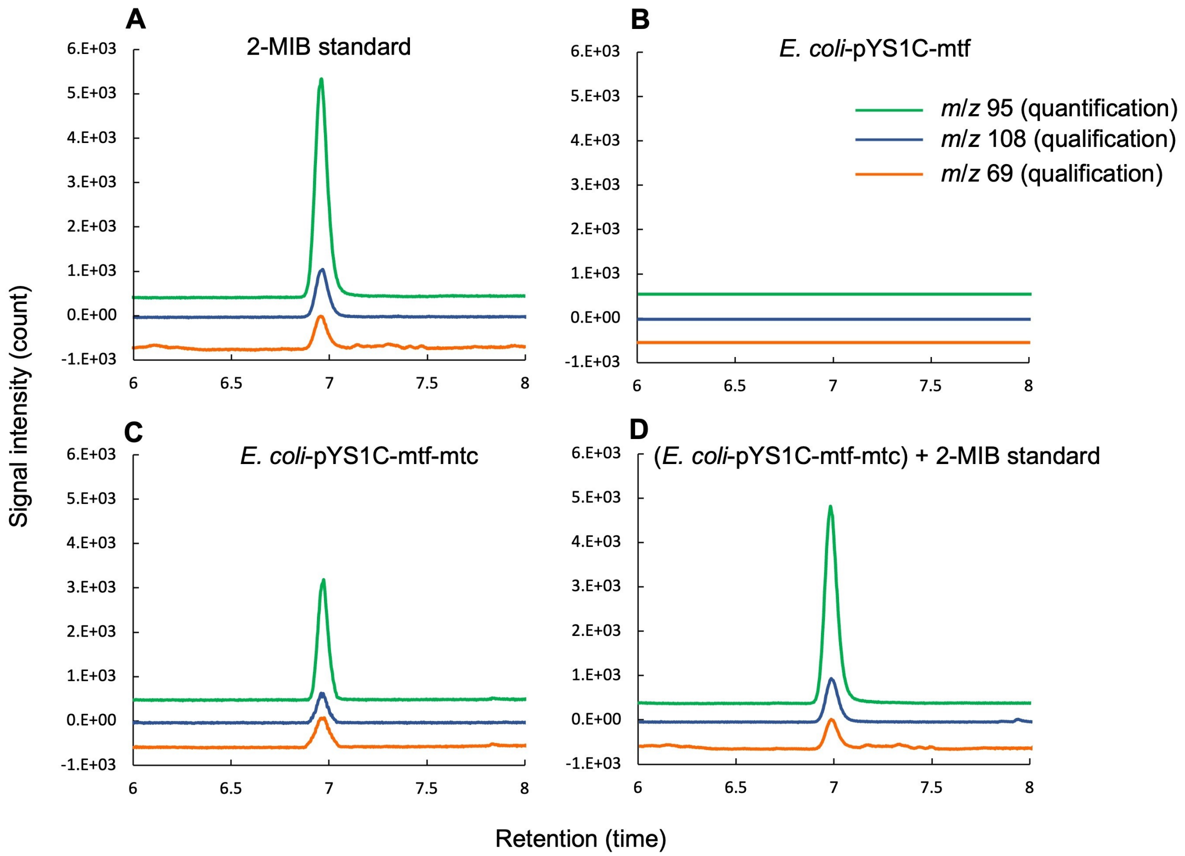

SUPPLEMENTARY FIGURE 2: Selected ion chromatograms of GC-MS. (A) 2-MIB standard, (B) *E. coli*-pYS1C-mtf, (C) *E. coli*-pYS1C-mtf-*mtc* and (D) *E. coli*-pYS1C-mtf-*mtc* spiked with 2-MIB standard were analyzed by GC-MS. Fragment *m*/*z* 95 was used for quantification and fragments *m*/*z* 108 and 69 were used for qualification of the 2-MIB peak.
